# Understanding the relationship between postpartum depression one month and six months after delivery and mother-infant bonding failure one-year after birth: results from the Japan Environment and Children's study (JECS)

**DOI:** 10.1017/S0033291719002101

**Published:** 2019-09-02

**Authors:** Haruka Kasamatsu, Akiko Tsuchida, Kenta Matsumura, Moeko Shimao, Kei Hamazaki, Hidekuni Inadera, the Japan Environment and Children's Study Group

**Affiliations:** 1 Toyama Regional Center for JECS, University of Toyama, Toyama, Japan; 2 Department of Public Health, Faculty of Medicine, University of Toyama, Toyama, Japan

**Keywords:** JECS, mother-infant bonding failure, mother-infant bonding, postpartum depression

## Abstract

**Background:**

Postpartum depression is a major mental health issue. It not only adversely affects the mother's quality of life, but also mother-infant bonding. However, the relationship between postpartum depression (at multiple points after childbirth) and mother-infant bonding failure one year after birth is not well understood. This study investigates the relationship between postpartum depression at 1-month and 6-month after birth and mother-infant bonding failure at 1 year after birth with a large cohort.

**Methods:**

Data from 83 109 mothers from the Japan Environment and Children's Study were analyzed. Mother-infant bonding 1-year after delivery was assessed using the Mother-to-Infant Bonding Scale Japanese version (MIBS-J). Postpartum depression was measured using the Edinburgh Postnatal Depression Scale (EPDS) at 1-month and 6-month after delivery. Twenty covariates during pregnancy and one month after delivery were controlled for deriving the odds ratios (ORs) describing postpartum depression to mother-infant bonding.

**Results:**

EPDS Total Score crude ORs and adjusted ORs against the MIBS-J Total Score at 1-month and 6-month after delivery were calculated. Crude ORs were 1.111 (95% CI 1.110–1.112) and 1.122 (95% CI 1.121–1.124) respectively. In the fully adjusted model, ORs were 1.088 (95% CI 1.086–1.089) and 1.085 (95% CI 1.083–1.087), respectively.

**Conclusions:**

This study demonstrated prospectively, in a large-scale cohort, that depression at multiple postpartum points, including associations with each EPDS and MIBS-J factors, may be a robust predictor of mother-infant bonding failure 1-year after birth.

## Introduction

Postpartum depression is a major mental health issue with average prevalence estimated at 13% worldwide (O'Hara and Swain, 1996), and around 10% in Japan (Yamashita *et al*., 2000). The cardinal symptoms of postpartum depression are anxiety, anhedonia, and depression (Cox *et al*., 1987; Kubota *et al*., 2014; Takehara *et al*., 2018). Its symptoms reduce a mother's capacity to be attuned to the needs of her infant, and distorted thoughts and reduced judgment affects the sensitivity of her responsiveness and, in turn, her parenting behaviors (Lefkovics *et al*., 2014). Further, postpartum depression increases the risk of child abuse and neglect, long-term impairment of the mother-child relationship, and psychiatric or learning disorders in children (Brockington, 2004). Mother-infant bonding is typically defined as the unidirectional positive emotions a mother has toward her infant and is seen as a significant motivator of parenting behaviors (Bicking Kinsey and Hupcey, 2013). Mother-infant bonding failure is, therefore, defined as a mother's indifference and alienation, or hostility and resentment toward her child (Kumar, 1997). Both longitudinal and cross-sectional research have demonstrated an association between postpartum depression and mother-infant bonding failure (Beck, 1995). Kerstis *et al*., found that maternal depression at 6-week postpartum had a significant impact on mother-infant bonding 6 months after delivery (Kerstis *et al*., 2016). However, few studies have measured postpartum depression at multiple points after birth and explained its relationship with mother-infant bonding failure one year after birth. A recent study investigated an association between postpartum depression at 4 weeks and mother-infant bonding after 1 year (O'Higgins *et al*., 2013). Unfortunately, these results are hard to generalize because of their limited sample size. Examining longitudinal associations such as these are hampered by the difficulties inherent in following large cohorts over time.

In our previous study, we evaluated the associations between bonding at 1-month postpartum and maternal depression at 1-month postpartum (Tsuchida *et al*., 2019). In the present study, together with these findings, we have evaluated the associations between bonding and maternal postpartum depression at 1-year postpartum, as around the age of one, children are going through an important period for the development of attachment (Takiguchi *et al*., 2015). A previous study has reported that children whose attachment development had been disturbed displayed various difficulties with cognition, behavior, social and emotional (Kay and Green, 2013). Healthy attachment comes to fruition when mothers and other primary caregivers give appropriate care when children demand it (Walsh, 2010). Thus, it is critical to evaluate the factors associated with bonding that contribute to the development of attachment. The present study focused on postpartum depression as a factor associated with bonding at 1-year postpartum. Although previous studies have evaluated postpartum depression at multiple time points and have investigated factors that are associated with bonding before and after 1-year postpartum, their samples were limited (Moehler *et al*., 2006; O'Higgins *et al*., 2013). Since any longitudinal study is faced with challenges in controlling the sample size and covariates, it remains unclear which symptoms that appear at what time point are associated with bonding at 1-year postpartum. Thus, this study prospectively assessed the association between postpartum depression at 1-month and 6-month after birth and mother-infant bonding failure at 1-year after birth in a large cohort from the Japan Environment and Children's Study (JECS) data.

## Methods

### Study design

The JECS is a government-funded birth cohort study conducted nationwide in Japan 2011–2014, registering 103 062 pregnancies that aims to evaluate the impact of environmental factors on children's health and development. Recruitment occurred across fifteen regional centers within Japan from 2011 to 2014, and detailed descriptions can be found elsewhere (Kawamoto *et al*., 2014; Michikawa *et al*., 2018).

Participant recruitment involved a face-to-face explanation of the survey to mothers, and written informed consent was obtained and recorded. The authors assert that all procedures contributing to this work comply with the ethical standards of the relevant national and institutional committees on human experimentation and with the Helsinki Declaration of 1975, as revised in 2008. All procedures involving human subjects for the JECS protocol were approved by the Institutional Review Board on Epidemiological Studies of the Ministry of the Environment (100910001), and the ethics committees of all participating institutions.

### Study data

**Fig. 1.**
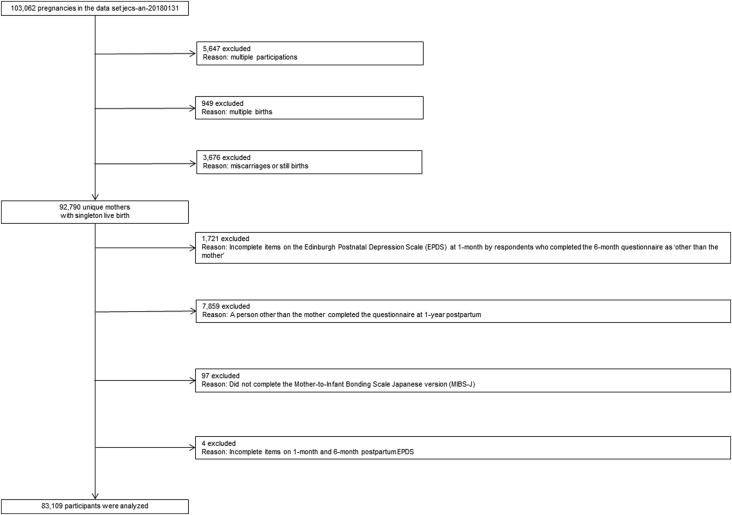
Participant flow diagram. See text for details.

The present study used the JECS datasets (jecs-an-20180131). This study included data from mothers with singleton, live births. Therefore, data from mothers having second and third births (5647), multiple births (949), and stillbirths/miscarriages (3676) were excluded. Other exclusion criteria were: (1) incomplete items on the Edinburgh Postnatal Depression Scale (EPDS) at 1 month by the respondents who completed the 6-month questionnaire as ‘other than the mother'; (2) a person other than the mother completing the questionnaire 1-year postpartum; (3) incomplete items on the Mother-to-Infant Bonding Scale Japanese version (MIBS-J); and (4) incomplete items on 1-month and 6-month postpartum EPDS. The final analytic dataset comprised 83 109 mothers (Fig. 1).

### Measurements

A self-report questionnaire was administered to the mothers during their first trimester, and second/third trimester in pregnancy, 1-month after delivery, 6-month after delivery, and 1-year after delivery to collect demographics, medical and obstetric history, physical and mental health issues, lifestyle factors, occupation, and socioeconomic status.

### Outcomes

The outcome variable was mother-infant bonding 1-year after delivery. Mother-infant bonding was assessed using the Mother-to-Infant Bonding Scale Japanese version (MIBS-J) (Yoshida *et al*., 2012). The MIBS-J consists of 10 items, answered using a four-point scale (0, not at all; 1, slightly, some of the time; 2, very much so, some of the time; and 3, very much so, most of the time). Some items are reverse scored to reduce response bias. The absolute score ranges from 0 to 30. The higher the MIBS-J score, the worse the mother-infant bonding. The MIBS-J has two subscales: Lack of Affection and Anger and Rejection. Both subscales included four items each (Lack of Affection: Items 1, 6, 8 and 10; Anger and Rejection: Items 2, 3, 5, and 7) (Yoshida *et al*., 2012). It is essential in clinical practice to understand both factor scores and the total score to ensure that no women who need support are overlooked (Kitamura *et al*., 2013). Therefore, both the total scores and subscales were analyzed here.

### Exposure

The main explanatory variable was postpartum depression at 1-month and 6-month after delivery. Postpartum depression was assessed using the Japanese version of the Edinburgh Postnatal Depression Scale (EPDS) (Okano *et al*., 1996). The EPDS consists of 10 items, answered using a four-point scale (total range = 0–30). A higher score indicates more depressive symptoms. In Japan, a score of 8/9 points is widely used as the cut-off point for defining postpartum depression (Hamazaki *et al*., 2018; Hirokawa *et al*., 2018; Okano *et al*., 1996). The EPDS has been expressed as a three-factor model for Anxiety, Anhedonia, and Depression (Kubota *et al*., 2014; Takehara *et al*., 2018). In this study the factors were classified as described below based on the three-factor models from previous studies (Kubota *et al*., 2014; Takehara *et al*., 2018) (Anxiety: EPDS Items 3, 4 and 5; Anhedonia: Items 1 and 2; Depression: Items 7, 9 and 10).

### Covariates

The covariates comprised the following variables: parity, maternal age, educational background (highest level of education attained), annual family income, marital status, physical activity, history of anxiety, history of depression, history of schizophrenia, history of dysautonomia, maternal depression [assessed by using the Kessler Psychological Distress Scale (K6)], diagnostic record of mental disorder (through data obtained during pregnancy), gestational weeks, infants' sex (measured at delivery), alcohol intake, smoking status, frequency that the respondent's partner takes care, feeding method, intensity and frequency of infants' crying, infants' anomaly (measured at 1-month postpartum).

### Statistical analysis

Descriptive statistics were used to present participants' demographic characteristics. The means and SD were calculated for all continuous variables used in this study, including the overall scores and subscale scores for the MIBS-J and EPDS, as aforementioned.

This study handled numerous covariates, which were categorized according to usual medical practice in Japan and/or referring to previous studies (Miyake *et al*., 2006; Hamazaki *et al*., 2018). The following covariates were included: parity (nullipara or multipara), maternal age (continuous years), educational background (highest level of education attained: high school and under, technical/vocational or other, or graduate and above), annual family income (<4 million, 4–6 million, or >6 million Japanese Yen), marital status (married, single/never married, or divorced/widowed), physical activity [continuous metabolic equivalent of tasks (METs), hours/day], history of anxiety (yes or no), history of depression (yes or no), history of schizophrenia (yes or no), history of dysautonomia (yes or no), maternal depression (total K6 scores), gestational weeks (continuous weeks), infant's sex (male or female), diagnostic record of mental disorder (yes or no), alcohol intake (never drinker, ex drinker, or current drinker), smoking status (never smoker, ex-smoker, or current smoker), frequency that the respondent's partner takes care of the infant (always, sometimes, seldom, and never), feeding method (breastfeeding only, mixed feeding, or infant formula only), infant's anomaly (yes or no), intensity and frequency of infant's crying (quite often and long, sometimes and short, or hardly).

Generalized Linear Model (GLM) analysis set logit as a link function, and after transforming the MIBS-J raw score into a ratio value (i.e. dividing the total and subscale scores by 30 and 12, respectively), the association between mother-infant bonding and postpartum depression was examined, adjusting for all other covariates outlined above. For the 6-month analysis, we first adjusted for these same previously mentioned covariates and the total EPDS score 1-month after birth. The GLM also examined the association between the two MIBS-J subscales (Lack of Affection, Anger and Rejection) and three EPDS subscales (Anxiety, Anhedonia, and Depression). Model 1 adjusted for the three EPDS subscales. Model 2 adjusted for the EPDS subscales and the previously listed covariates. For the 6-month analysis, we added all of the EPDS subscales from the 1-month after birth as covariates to both Model 1 and Model 2.

As an additional analysis, this study compared the MIBS-J total scores for four groups, which were classified based on their EPDS Total Scores at 1-month and 6-month after delivery using the EPDS cut-off point (setting at 8/9). The four groups included those with a score of <9 at both time points, those with scores of ⩾9 at 1-month and <9 at 6-month, those with a score of <9 at 1-month and ⩾9 at 6-month, and those with scores of ⩾9 at both time points.

Two-tailed *p* values < 0.05 were considered statistically significant. Data analysis used SAS version 9.4 software (SAS Institute Inc., Cary, NC).

### Missing data

For the 83 109 pregnancies included in this study, the missing data for most covariates was less than 2%; physical activity during pregnancy (5.4%), and annual household income (7.5%) were the only covariates that were higher. For the exposure measures, each item of the EPDS at 1- month and 6-month had missing data of ⩽0.92% (max *n* = 765), ⩽1.81% (max *n* = 1501), respectively. For the outcome measures, each item of MIBS-J had ⩽0.16% (max *n* = 135). Overall, 15 133 mothers (18.2%) had at least one missing value. Imputation using chained equations (van Buuren, 2007) was conducted to get ten imputed data sets. All data were imputed simultaneously regardless of measured time points. Auxiliary variables related to analyzed variables ⩾0.40 multiple correlation coefficients were included to preserve the assumption of ‘missing at random.’

### Sensitivity analysis

Odds ratios (ORs) were calculated from cases at 1-month after birth and 6-month after birth data sets (1-month, *n* = 81 141 and 6-month, *n* = 79 022) with those from the multiply imputed data set (*n* = 83 109) to assess the difference between the strategies addressing missing values.

## Results

Table 1 shows the characteristics of participants. The participants' mean MIBS-J total was 2.0 (s.d. = 2.3). The EPDS total at 1-month after birth was 5.1 (s.d. = 3.5), and at 6-month after birth was 4.6 (s.d. = 3.4).

**Table 1.** Demographic and obstetric characteristics of participants (*N* = 83 109)

Characteristics	Mean/N	(SD/%)
EPDS score at 1 month after birth, mean (s.d.)		
Total	5.1	(3.5)
Anxiety	2.9	(1.9)
Anhedonia	0.2	(0.6)
Depression	0.3	(0.9)
EPDS score at 6 months after birth, mean (s.d.)		
Total	4.6	(3.4)
Anxiety	2.6	(1.9)
Anhedonia	0.1	(0.4)
Depression	0.4	(1.0)
Bonding score at 1 year after birth, mean (s.d.)		
Total	2.0	(2.3)
Lack of Affection	0.6	(1.0)
Anger and Rejection	1.2	(1.4)
Maternal age at 1 month after birth, mean (s.d.)	31.4	(5.0)
Previous deliveries, *n* (%)		
Nullipara	36 889	(44.4)
Multipara	46 179	(55.6)
Highest educational level, *n* (%)		
Junior high school or high school	28 525	(34.7)
Technical junior college, technical/ vocational College or associate degree	35 082	(42.7)
Bachelor's degree, postgraduate degree	18 529	(22.6)
Annual household income (JPY), *n* (%)		
<4 million	30 204	(39.3)
4–6 million	25 703	(33.4)
>6 million	21 006	(27.3)
Marital status, *n* (%)		
Married (including common law marriage)	78 795	(95.8)
Single (never married)	2860	(3.5)
Divorced/widowed	631	(0.8)
Alcohol intake at 1 month after birth, *n* (%)		
Never drank	75 574	(91.6)
Ex-drinkers who quit before pregnancy	3656	(4.4)
Current drinker	3269	(4.0)
Smoking status at 1 month after birth, *n* (%)		
Never a smoker	49 143	(59.6)
Ex-smoker	30 247	(36.7)
Current smoker	3028	(3.7)
Physical activity during pregnancy (mets h/day), mean (s.d.)	3.9	(8.2)
History of depression, yes (%)	2463	(3.0)
History of anxiety disorder, yes (%)	2273	(2.8)
History of schizophrenia, yes (%)	133	(0.2)
History of dysautonomia, yes (%)	3014	(3.7)
Diagnostic record of mental disorder during pregnancy, yes (%)	632	(0.8)
Total K6 score during pregnancy, mean (s.d.)	3.4	(3.7)
Frequency that the respondent's partner takes care of the baby at 1 month after birth, *n* (%)		
Always	31 547	(38.7)
Sometimes	38 046	(46.7)
Seldom	9312	(11.4)
Never	2608	(3.2)
Feeding method during the 1 month after birth, *n* (%)		
Breastfeeding only	34 936	(42.4)
Mixed feeding	46 408	(56.3)
Infant formula only	1087	(1.3)
Gestational weeks, mean (s.d.)	38.8	(1.6)
Infant's anomaly, yes (%)	1723	(2.1)
Infant's gender, *n* (%)		
Male	42 661	(51.3)
Female	40 448	(48.7)
Intensity and frequency of infants' crying, *n* (%)		
Quite often and long	14 092	(17.1)
Sometimes and short	64 353	(78.3)
Hardly	3761	(4.6)

Mets h/day, metabolic equivalent of a task measured as the number of hours per day.

Out of 92 790 mothers, 9681 were excluded from analysis (Fig. 1). Compared with the 9681 non-participants, a large proportion of the 83 109 was primipara, educated (more than technical junior college, technical/vocational college or associate degree), had an annual household income of JPY4 million or more, and was married. A smaller proportion of the participants drank and smoked at 1-month after delivery, had a lower K6 score during pregnancy, had infants with anomalies, and were more likely to be older. There were no material differences between the 9681 non-participants excluded from analysis and the 83 109 participants who were analyzed with regard to the distribution of other covariates.

Table 2 shows the EPDS total score crude ORs and adjusted ORs at 1-month and 6-month after delivery *v.* the MIBS-J Total Score. Significant associations of the EPDS Total Score and the MIBS-J Total Score were observed both at 1-month and 6-month after delivery, which were also observed after adjusting by including other covariates.

**Table 2.** Results of generalized linear model between postpartum depression and bonding

	Total bonding score	
	ORs	(95% CI)
Total EPDS score		
One-month after birth		
Crude	1.111	(1.110–1.112)
Adjusted^a^	1.088	(1.086–1.089)
Six-month after birth		
Crude	1.122	(1.121–1.124)
Adjusted^b^	1.085	(1.083–1.087)

CI, Confidence Interval; ORs, Odds ratios

a Covariates that were adjusted for include: parity, maternal age, educational background (i.e. highest level of education attained), annual family income, marital status, physical activity, history of anxiety, history of depression, history of schizophrenia, history of dysautonomia, maternal depression (i.e. total K6 scores), gestational weeks, infants' sex, diagnostic record of mental disorder, alcohol intake, smoking status, frequency that the respondent's partner takes care, feeding method, infant's anomaly, intensity and frequency of infants' crying.

b The same covariates were adjusted for in Model 2 as were list above in footnote ‘a' and the total EPDS score 1-month after birth was added as a covariate.

Table 3 shows the crude ORs and adjusted ORs for each of the EPDS factors (Anxiety, Anhedonia, and Depression) at 1 month and 6 months after delivery *v.* each of the MIBS-J factors (Lack of Affection, Anger and Rejection). The EPDS factors (Anxiety, Anhedonia, and Depression) at 1-month and 6-month after delivery were significantly associated with each of the MIBS-J factors (Lack of Affection, Anger and Rejection), respectively. It was also observed after adjusting the covariates (Model 1 and 2). Lack of Affection showed a stronger association with Anhedonia than any other EPDS subscales, and Anger and Rejection showed a stronger association with Anxiety than any other EPDS subscales.

**Table 3.** Results of generalized linear model between postpartum depression and bonding by each subscale

	Bonding sub-score			
	Lack of affection		Anger and rejection	
	ORs	(95% CI)	ORs	(95% CI)
**EPDS sub-score**				
*One-month after birth*				
Anxiety				
Crude	1.142	(1.137–1.147)	1.246	(1.242–1.250)
Model 1	1.082	(1.076–1.088)	1.205	(1.201–1.210)
Model 2	1.069	(1.063–1.075)	1.154	(1.149–1.159)
Anhedonia				
Crude	1.441	(1.426–1.457)	1.380	(1.368–1.392)
Model 1	1.265	(1.249–1.282)	1.078	(1.067–1.090)
Model 2	1.224	(1.208–1.240)	1.061	(1.049–1.072)
Depression				
Crude	1.230	(1.222–1.238)	1.286	(1.280–1.293)
Model 1	1.074	(1.064–1.083)	1.082	(1.075–1.089)
Model 2	1.057	(1.047–1.067)	1.051	(1.043–1.058)
*Six-month after birth*				
Anxiety				
Crude	1.170	(1.164–1.175)	1.272	(1.268–1.277)
Model 1^a^	1.080	(1.073–1.087)	1.149	(1.143–1.154)
Model 2^b^	1.080	(1.073–1.087)	1.147	(1.142–1.153)
Anhedonia				
Crude	1.591	(1.567–1.615)	1.534	(1.515–1.553)
Model 1^a^	1.189	(1.167–1.211)	1.061	(1.045–1.077)
Model 2^b^	1.172	(1.150–1.194)	1.075	(1.059–1.092)
Depression				
Crude	1.244	(1.236–1.252)	1.305	(1.298–1.311)
Model 1^a^	1.060	(1.050–1.071)	1.064	(1.056–1.071)
Model 2^b^	1.066	(1.055–1.076)	1.055	(1.047–1.063)

CI, Confidence Interval; ORs, Odds ratios.

Model 1: After adjusting for all other EPDS subscales.

Model 2: After adjusting for all other EPDS subscales, as well as parity, maternal age, educational background (highest level of education attained), annual family income, marital status, physical activity, history of anxiety, history of depression, history of schizophrenia, history of dysautonomia, maternal depression (total K6 scores), gestational weeks, infants' sex, diagnostic record of mental disorder, alcohol intake, smoking status, frequency that the respondent's partner takes care, feeding method, infant's anomaly, intensity and frequency of infants' crying.

a Covariates included the same factors as described for Model 1 and all EPDS subscales 1-month after birth.

b Covariates included the same factors described for ‘Model 2’ and all EPDS subscales 1-month after birth.

**Fig. 2.**
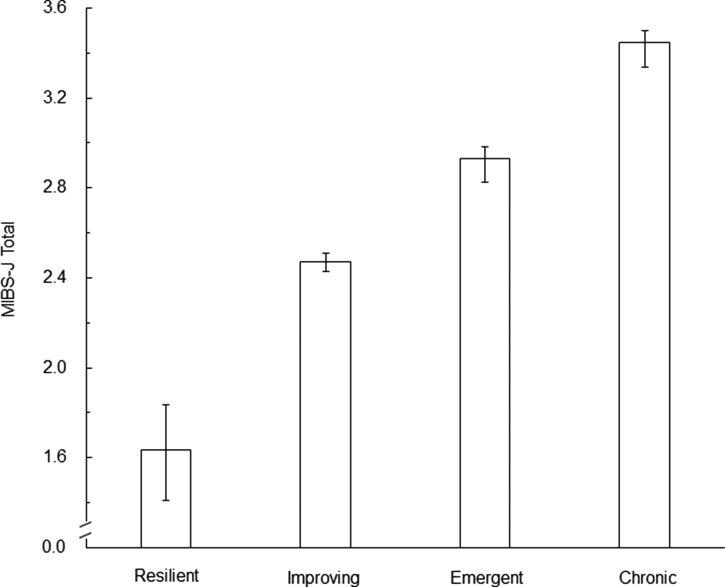
Comparing MIBS-J scores each group. *X*-axis represents group based on the scores after 1 month and 6 months after delivery. *Y*-axis represents the Mother-to-Infant Bonding Scale Japanese version (MIBS-J) total score. Bar represents means of MIBS-J total score. Error bar represents 95% confidence intervals.

The EPDS cut-off point was set at 8/9, and the participants were classified into four groups based on their scores 1-month and 6-month after delivery. Those with a score of <9 at both time points were termed the Resilient Group (80.8%); those with scores of ⩾9 at 1-month and <9 at 6-month, the Improving Group (7.7%); those with a score of <9 at 1-month and ⩾9 at 6-month, the Emergent Group (5.1%); and those with scores of ⩾9 at both time points the Chronic Group (6.5%). The results indicated that the mean score on the MIBS-J for the Resilient Group was 1.64 [95% CI(1.43–1.86)], Improving Group was 2.47 [95% CI(2.43–2.51)], Emergent Group was 2.93 (95% CI(2.88–2.98)], and the Chronic Group was 3.45 [95% CI(3.39–3.50); Fig. 2].

Results of sensitivity analyses using the complete case dataset were not meaningfully different from the initial results (data not shown).

## Discussion

This study provides evidence of a longitudinal association between depression symptoms at two time points postpartum (at 1-month and 6-month after delivery) and mother-infant bonding failure at 1-year after birth using a large-scale cohort sample. While previously the longitudinal association between postpartum depression and mother-infant bonding failure has been demonstrated (O'Higgins *et al*., 2013; Kerstis *et al*., 2016), it has not evaluated at 1-year postpartum using a large-scale cohort. The results of this study indicate that postpartum depression is associated with mother-infant bonding failure, thus, bolstering the findings of previous studies (O'Higgins *et al*., 2013; Kerstis *et al*., 2016).

This study identified the relationship between the EPDS subscales at 1 month and 6 months after delivery and MIBS-J subscales one year after birth. While both EPDS and MIBS-J comprise multiple factors(Yoshida *et al*., 2012; Kubota *et al*., 2014; Takehara *et al*., 2018), few studies have investigated the association between each factor of postpartum depression and mother-infant bonding failure. This study demonstrated that all EPDS subscales at 1-month and 6-month postpartum were significantly associated with all MIBS-J subscales at 1-year. Furthermore, these results showed that Anhedonia had a stronger association with Lack of Affection, and Anxiety had a stronger association with Anger and Rejection than the other EPDS subscales at both 1-month and 6-month after delivery.

Each covariate of depression at 1-month and 6-month after delivery, even after adjustment, demonstrated a robust association with mother-infant bonding failure at 1 year after birth. This is not surprising given that mother-infant bonding depends on the feelings and emotions a mother feels towards her own infant (Bicking Kinsey and Hupcey, 2013). Arguably, the emotional aspects of the postpartum depression, coupled with the physical fatigue and insomnia, affect the mother's feelings and emotions towards the infant.

The ORs of depression at 1-month and 6-month after delivery for mother-infant bonding failure at 1-year after birth were not significantly different. Generally, the timing of 1 month after delivery overlaps with a medical examination and neonatal visit, so it is easy for medical staff to interact with both the mother and infant. Identification of depression at the early postpartum stage may be tied in with the subsequent mental health of the mother and the mother-infant relationship. Although 6-month after delivery is in close proximity in time to the 1-year after delivery, our results suggest that the effects of depression at 1-month and 6-month after delivery on mother-infant bonding failure are almost the same. Therefore, identifying depression at the early postpartum stage may be a sufficient predictor for mother-infant bonding failure at 1-year after birth.

There are multiple prognostic factors for mother-infant bonding failure (Edhborg *et al*., 2011), so postpartum depression is not the sole predictor. This study investigated the association between postpartum depression and mother-infant bonding failure by adjusting for other covariates. The adjusted model compared with the crude data changed the ORs by 0.023 at 1-month after delivery and 0.037 at 6-month after delivery. Even with the covariates, depression at 1-month and 6-month after delivery are robust predictors of mother-infant bonding failure. This study also used a history of pre-pregnancy maternal depression and depression during pregnancy (K6) as covariates, which enabled the investigation to focus on postpartum depression. Based on the aforementioned information, it could be said that depression at 1-month and 6-month after delivery has a prospective association with mother-infant bonding failure at 1-year after birth, even when the effect of the covariates is taken into consideration.

This study confirmed that all of the EPDS subscales predicted all of the MIBS-J subscales at 1-year after birth and suggests that even in cases where only one symptom of postpartum depression appears, symptoms of mother-infant bonding failure 1-year after delivery may occur. Furthermore, although there were differences in the maximum scores across the EPDS subscales, our results showed that Anhedonia showed a stronger association with Lack of Affection than did the other EPDS subscales both 1-month and 6-month after delivery. This could be understood as a manifestation of the core depressive symptom of losing joy and interest (i.e. Anhedonia) in the form of the loss of attachment to children and loss of delight with childrearing during interactions with children.

In addition, Anxiety showed stronger association with Anger and Rejection than did other EPDS subscales both 1-month and 6-month after delivery. Anhedonia and depression are considered as core depressive symptoms (American Psychiatric Association, 2013), whereas Anxiety is considered a symptom particular to postpartum depression (Okano *et al*., 1996; Kubota *et al*., 2014). It is worth noting that Anxiety, a symptom of postpartum depression, is correlated more strongly with anger and negative emotions than the core depressive symptoms. A previous study reported that anger could trigger aggressive behavior (Graham *et al*., 2001), and worsened symptoms may lead to serious child abuse. In the clinical practice of screening and developing an intervention for postpartum depression, providing care focused on symptoms of anxiety along with the core depressive symptoms, may have a positive impact on mothers' emotions toward their children and their childrearing performance.

Comparing MIBS-J scores at 1-year after birth, indicated that scores at 1-year after birth were significantly higher in the Improving, Emergent, and Chronic Groups than in the Resilient Group. This suggests a higher risk of mother-infant bonding failure if the EPDS cut-off point is exceeded at either 1-month or 6-month after delivery. This is aligned with the results of the GLM and reinforces the longitudinal association between postpartum depression and mother-infant bonding failure. Interestingly, the mean MIBS-J score at 1-year after birth was significantly lower in the Improving Group compared to the Emergent and Chronic Groups. This suggests that if depression develops, improving depression at an early postpartum stage would reduce the risk of mother-infant bonding failure at 1-year after birth. The Improving Group could be considered as a group that had spontaneous recovery from depression, but proactively targeting depression through intervention and support could significantly reduce the risk of or even prevent mother-infant bonding failure.

### Strengths and limitations

The benefit of this study is that it has demonstrated prospectively using a large- scale cohort that depression at multiple postpartum points may be a predictor of mother-infant bonding failure at 1-year after birth. In addition, this study clarified the associations between each EPDS and MIBS-J subscale. Moreover, the results indicated that mothers who recovered from postpartum depression at an earlier period might have a reduced risk of experiencing bonding failure symptoms at 1-year postpartum compared to those who did not. The advantage of the present study is its ability to demonstrate these results with a much larger sample size than those used in previous studies, which will make greater precision in estimating effect sizes and a capacity to run meaningful controls for numerous covariates.

This study has a few limitations. First, this study evaluated postpartum depression using self-reported EPDS questionnaires, which are more prone to social desirability bias (Gorman *et al*., 2004). For example, it is known that Japanese people under-evaluated their depressive symptoms on self- report measures compared to those in other countries (Okano *et al*., 1996). Accordingly, the prevalence of postpartum depression may differ in this study from rates obtained using clinical diagnostic criteria. In addition, this study was unable to evaluate maternal depression at 1-year postpartum as we could not obtain data on the EPDS at 1-year postpartum. If this study was able to assess depression at 1-year after delivery, it would be possible to more carefully assess the association of postpartum depression and mother-infant bonding failure over time. Second, mother-infant bonding failure itself is difficult to define as disease or illness, as a mother's indifference and alienation, or hostility and resentment toward her child do not fit comfortably with the concept of disease or illness (Brockington, 2004). Investigations have been conducted on the MIBS-J cut-off point (Matsunaga *et al*., 2017), but the natural course of mother-infant bonding failure is still unclear. The cut-off point may change depending upon the target group and the measurement timing; so careful consideration is essential before applying this to all patient populations. Measurement methods include the Stafford interview, in addition to self-administered questionnaires (Brockington *et al*., 2017). Since there is no standardized definition, it is essential to adopt a more multifaceted approach, including a number of objective indicators, to ascertain the condition of mother-infant bonding failure. Third, several findings have concluded that one of the main predictors for mother-infant bonding failure is maternal bonding with the fetus (Ohara *et al*., 2017). The JECS data did not measure bonding with the fetus during pregnancy, so this could not be considered as a covariate. Lastly, it is not possible to draw conclusions about causality between postpartum depression and subsequent bonding failure since this study is observational. The demonstration of a causal relationship requires an intervention approach with mothers of depressive state. Moreover, a previous study reported that bonding failure predicted depressive mood during pregnancy and 5 days after delivery (Ohara *et al*., 2017). It has also been demonstrated that even if postpartum depression is improved, mother-infant bonding failure can persist (Moehler *et al*., 2006), and mother-infant bonding failure is known to occur also without postpartum depression (Righetti-Veltema *et al*., 2002; Klier, 2006).

Despite these limitations, this study was able to successfully explain the association between postpartum depression and bonding failure prospectively using a large cohort sample and adjusting for many covariates. Our findings have shown that postpartum depression can be a robust predictor of bonding failure 1-year after delivery and strengthen the notion that depression must be treated in pregnant and postpartum women worldwide (Netsi *et al*., 2018; Curry *et al*., 2019). Additionally, this study suggested that early intervention for postpartum depression may reduce the risk of bonding failure 1-year after delivery. For postpartum depression, counseling interventions, such as cognitive behavioral therapy and interpersonal therapy, have been recommended, and their effects have been proven (Curry *et al*., 2019). In the future, these interventions for postpartum depression are expected to inhibit maternal rejection and harm toward children.

## Conclusions

Our analyses of data from a large-scale cohort study highlighted that women who display depressive symptoms at 1-month or 6-month after delivery are more likely to exhibit bonding failure symptoms at 1-year postpartum, which is a critical period for children's attachment development. Furthermore, our analyses demonstrated that all symptoms of postpartum depression are associated with all symptoms of bonding failure and revealed the type of association for each symptom. For those who displayed symptoms of postpartum depression, recovering from the symptoms at an early stage of the infants' development decreased the risk of bonding failure 1-year after delivery. Taken together, this study suggests that early interventions for postpartum depression may play a critical role in reducing mother-infant bonding failure.

## Appendix

Members of the JECS (principal investigator, Michihiro Kamijima) as of 2019: Shin Yamazaki (National Institute for Environmental Studies, Tsukuba, Japan), Yukihiro Ohya (National Center for Child Health and Development, Tokyo, Japan), Reiko Kishi (Hokkaido University, Sapporo, Japan), Nobuo Yaegashi (Tohoku University, Sendai, Japan), Koichi Hashimoto (Fukushima Medical University, Fukushima, Japan), Chisato Mori (Chiba University, Chiba, Japan), Shuichi Ito (Yokohama City University, Yokohama, Japan), Zentaro Yamagata (University of Yamanashi, Chuo, Japan), Hidekuni Inadera (University of Toyama, Toyama, Japan), Michihiro Kamijima (Nagoya City University, Nagoya, Japan), Takeo Nakayama (Kyoto University, Kyoto, Japan), Hiroyasu Iso (Osaka University, Suita, Japan), Masayuki Shima (Hyogo College of Medicine, Nishinomiya, Japan), Youichi Kurozawa (Tottori University, Yonago, Japan), Narufumi Suganuma (Kochi University, Nankoku, Japan), Koichi Kusuhara (University of Occupational and Environmental Health, Kitakyushu, Japan), and Takahiko Katoh (Kumamoto University, Kumamoto, Japan).

## References

[ref1] American Psychiatric Association (2013) Diagnostic and Statistical Manual of Mental Disorders, 5th Edn. Washington, DC.

[ref2] Beck CT (1995) The effects of postpartum depression on maternal-infant interaction: a meta-analysis. Nursing Research 44, 298–304. 7567486

[ref3] Bicking Kinsey C and Hupcey JE (2013) State of the science of maternal-infant bonding: a principle-based concept analysis. Midwifery 29, 1314–1320. 23452661 10.1016/j.midw.2012.12.019 PMC3838467

[ref4] Brockington I (2004) Postpartum psychiatric disorders. Lancet 363, 303–310. 14751705 10.1016/S0140-6736(03)15390-1

[ref5] Brockington I, Chandra P, Bramante A, Dubow H, Fakher W, Garcia-Esteve L, Hofberg K, Moussa S, Palacios-Hernandez B, Parfitt Y and Shieh PL (2017) The stafford interview : a comprehensive interview for mother-infant psychiatry. Archives of Womens Mental Health 20, 107–112. 10.1007/s00737-016-0683-8 PMC5237445 27778149

[ref6] Cox JL, Holden JM and Sagovsky R (1987) Detection of postnatal depression. Development of the 10-item Edinburgh Postnatal Depression Scale. The British Journal of Psychiatry 150, 782–786. 3651732 10.1192/bjp.150.6.782

[ref7] Curry SJ, Krist AH, Owens DK, Barry MJ, Caughey AB, Davidson KW, Doubeni CA, Epling Jr. JW, Grossman DC, Kemper AR, Kubik M, Landefeld CS, Mangione CM, Silverstein M, Simon MA , Tseng CW and Wong JB (2019) Interventions to prevent perinatal depression: US preventive services task force recommendation statement. JAMA 321, 580–587. 30747971 10.1001/jama.2019.0007

[ref8] Edhborg M, Nasreen HE and Kabir ZN (2011) Impact of postpartum depressive and anxiety symptoms on mothers' emotional tie to their infants 2–3 months postpartum: a population-based study from rural Bangladesh. Archives of Women's Mental Health 14, 307–316. 10.1007/s00737-011-0221-7 21626173

[ref9] Gorman LL, O'Hara MW, Figueiredo B, Hayes S, Jacquemain F, Kammerer MH, Klier CM, Rosi S, Seneviratne G and Sutter-Dallay AL (2004) Adaptation of the structured clinical interview for DSM-IV disorders for assessing depression in women during pregnancy and post-partum across countries and cultures. The British Journal of Psychiatry. Supplement 46, s17–s23. 14754814 10.1192/bjp.184.46.s17

[ref10] Graham S, Weiner B, Cobb M and Henderson T (2001) An attributional analysis of child abuse among Low-income African American Mothers. Journal of Social and Clinical Psychology 20, 233–257.

[ref11] Hamazaki K, Takamori A, Tsuchida A, Kigawa M, Tanaka T, Ito M, Adachi Y, Saito S, Origasa H, Inadera H and the Japan Environment and Children's Study Group (2018) Dietary intake of fish and n-3 polyunsaturated fatty acids and risks of perinatal depression: The Japan Environment and Children's Study (JECS). Journal of Psychiatric Research 98, 9–16. 29253720 10.1016/j.jpsychires.2017.11.013

[ref12] Hirokawa K, Kimura T, Ikehara S, Honjo K, Sato T, Ueda K, Iso H and the Japan Environment and Children's Study Group (2018) Associations between broader autism phenotype (BAP) and maternal attachment are moderated by maternal postpartum depression when infants are one month old: a prospective study of the Japan environment & children's study. Journal of Affective Disorders 243, 485–493. 30273887 10.1016/j.jad.2018.09.060

[ref13] Kawamoto T, Nitta H, Murata K, Toda E, Tsukamoto N, Hasegawa M, Yamagata Z, Kayama F, Kishi R, Ohya Y, Saito H, Sago H, Okuyama M, Ogata T, Yokoya S, Koresawa Y, Shibata Y, Nakayama S, Michikawa T, Takeuchi A, Satoh H and the Japan Environment and Children's Study Group (2014) Rationale and study design of the Japan environment and children's study (JECS). BMC Public Health 14, 25. 24410977 10.1186/1471-2458-14-25 PMC3893509

[ref14] Kay C and Green J (2013) Reactive attachment disorder following early maltreatment: systematic evidence beyond the institution. Journal of Abnormal Child Psychology 41, 571–581. 23250477 10.1007/s10802-012-9705-9

[ref15] Kerstis B, Aarts C, Tillman C, Persson H, Engstrom G, Edlund B, Ohrvik J, Sylven S and Skalkidou A (2016) Association between parental depressive symptoms and impaired bonding with the infant. Archives of Women's Mental Health 19, 87–94. 10.1007/s00737-015-0522-3 25854998

[ref16] Kitamura T, Takegata M, Haruna M, Yoshida K, Yamashita H, Murakami M and Goto Y (2013) The mother-infant bonding scale: factor structure and psychosocial correlates of parental bonding disorders in Japan. Journal of Child and Family Studies 24, 393–401.

[ref17] Klier CM (2006) Mother-infant bonding disorders in patients with postnatal depression: the postpartum bonding questionnaire in clinical practice. Archives of Women's Mental Health 9, 289–291. 10.1007/s00737-006-0150-z 16937312

[ref18] Kubota C, Okada T, Aleksic B, Nakamura Y, Kunimoto S, Morikawa M, Shiino T, Tamaji A, Ohoka H, Banno, N., Morita T, Murase S, Goto S, Kanai A, Masuda T, Ando M and Ozaki N (2014) Factor structure of the Japanese version of the Edinburgh postnatal depression scale in the postpartum period. PLoS One 9, e103941. 25089523 10.1371/journal.pone.0103941 PMC4121230

[ref19] Kumar RC (1997) “Anybody's child”: severe disorders of mother-to-infant bonding. The British Journal of Psychiatry 171, 175–181. 9337956 10.1192/bjp.171.2.175

[ref20] Lefkovics E, Baji I and Rigo J (2014) Impact of maternal depression on pregnancies and on early attachment. Infant Mental Health Journal 35, 354–365. 25798487 10.1002/imhj.21450

[ref21] Matsunaga A, Takauma F, Tada K and Kitamura T (2017) Discrete category of mother-to-infant bonding disorder and its identification by the mother-to-infant bonding scale: a study in Japanese mothers of a 1-month-old. Early Human Development 111, 1–5. 28525876 10.1016/j.earlhumdev.2017.04.008

[ref22] Michikawa T, Nitta H, Nakayama SF, Yamazaki S, Isobe T, Tamura K, Suda E, Ono M, Yonemoto J, Iwai-Shimada M, Kobayashi Y, Suzuki G, Kawamoto T and the Japan Environment and Children's Study Group (2018) Baseline profile of participants in the Japan Environment and Children's Study (JECS). Journal of Epidemiology 28, 99–104. 29093304 10.2188/jea.JE20170018 PMC5792233

[ref23] Miyake Y, Sasaki S, Yokoyama T, Tanaka K, Ohya Y, Fukushima W, Saito K, Ohfuji S, Kiyohara C and Hirota Y (2006) Risk of postpartum depression in relation to dietary fish and fat intake in Japan: the Osaka Maternal and Child Health Study. Psychological Medicine 36, 1727–1735. 16938145 10.1017/S0033291706008701

[ref24] Moehler E, Brunner R, Wiebel A, Reck C and Resch F (2006) Maternal depressive symptoms in the postnatal period are associated with long-term impairment of mother-child bonding. Archives of Women's Mental Health 9, 273–278. 10.1007/s00737-006-0149-5 16937313

[ref25] Netsi E, Pearson RM, Murray L, Cooper P, Craske MG and Stein A (2018) Association of persistent and severe postnatal depression with child outcomes. JAMA Psychiatry 75, 247–253. 29387878 10.1001/jamapsychiatry.2017.4363 PMC5885957

[ref26] O'Hara MW and Swain AM (1996) Rates and risk of postpartum depression—a meta-analysis. International Review of Psychiatry 8, 37–54.

[ref27] Ohara M, Okada T, Kubota C, Nakamura Y, Shiino T, Aleksic B, Morikawa M, Yamauchi A, Uno Y, Murase S, Goto S, Kanai A, Masuda T, Ando M and Ozaki N (2017) Relationship between maternal depression and bonding failure: a prospective cohort study of pregnant women. Psychiatry and Clinical Neurosciences 71, 733–741. 28556440 10.1111/pcn.12541

[ref28] O'Higgins M, Roberts IS, Glover V and Taylor A (2013) Mother-child bonding at 1 year; associations with symptoms of postnatal depression and bonding in the first few weeks. Archives of Women's Mental Health 16, 381–389. 10.1007/s00737-013-0354-y 23604546

[ref29] Okano T, Murata M, Masuji F, Tamaki R, Nomura J, Miyaoka H and Kitamura T (1996) Validation and reliability of Japanese version of EPDS [Japanese Article]. Archives of Psychiatry and Diagnosis in Clinical Evaluation 7, 525–533.

[ref30] Righetti-Veltema M, Conne-Perreard E, Bousquet A and Manzano J (2002) Postpartum depression and mother-infant relationship at 3 months old. Journal of Affective Disorders 70, 291–306. 12128241 10.1016/s0165-0327(01)00367-6

[ref31] Takehara K, Tachibana Y, Yoshida K, Mori R, Kakee N and Kubo T (2018) Prevalence trends of pre- and postnatal depression in Japanese women: a population-based longitudinal study. Journal of Affective Disorders 225, 389–394. 28846961 10.1016/j.jad.2017.08.008

[ref32] Takiguchi S, Fujisawa TX, Mizushima S, Saito DN, Okamoto Y, Shimada K, Koizumi M, Kumazaki H, Jung M, Kosaka H, Hiratani M, Ohshima Y, Teicher MH and Tomoda A (2015) Ventral striatum dysfunction in children and adolescents with reactive attachment disorder: functional MRI study. BJPsych Open 1, 121–128. 27703736 10.1192/bjpo.bp.115.001586 PMC4995568

[ref33] Tsuchida A, Hamazaki K, Matsumura K, Miura K, Kasamatsu H, Inadera H and the Japan Environment and Children's Study Group (2019) Changes in the association between postpartum depression and mother-infant bonding by parity: longitudinal results from the Japan Environment and Children's Study. Journal of Psychiatric Research 110, 110–116. 30616158 10.1016/j.jpsychires.2018.11.022

[ref34] van Buuren S (2007) Multiple imputation of discrete and continuous data by fully conditional specification. Statistical Methods in Medical Research 16, 219–242. 17621469 10.1177/0962280206074463

[ref35] Walsh J (2010) Definitions matter: if maternal-fetal relationships are not attachment, what are they? Archives of Women's Mental Health 13, 449–451. 10.1007/s00737-010-0152-8 20217158

[ref36] Yamashita H, Yoshida K, Nakano H and Tashiro N (2000) Postnatal depression in Japanese women. Detecting the early onset of postnatal depression by closely monitoring the postpartum mood. Journal of Affective Disorders 58, 145–154. 10781704 10.1016/s0165-0327(99)00108-1

[ref37] Yoshida K, Yamashita H, Conroy S, Marks M and Kumar C (2012) A Japanese version of Mother-to-Infant Bonding Scale: factor structure, longitudinal changes and links with maternal mood during the early postnatal period in Japanese mothers. Archives of Women's Mental Health 15, 343–352. 10.1007/s00737-012-0291-1 PMC3443344 22733162

